# Editorial: Precision of minimally invasive surgery for intracerebral hemorrhage treatment

**DOI:** 10.3389/fneur.2022.996394

**Published:** 2022-08-30

**Authors:** Zhouping Tang, John Zhang, Qiang Dong, Guofeng Wu, Yu Hasegawa, Christopher Paul Kellner

**Affiliations:** ^1^Tongji Hospital, Tongji Medical College, Huazhong University of Science and Technology, Wuhan, China; ^2^School of Medicine, Loma Linda University, Loma Linda, CA, United States; ^3^Huashan Hospital, Shanghai Medical College, Fudan University, Shanghai, China; ^4^Affiliated Hospital of Guizhou Medical University, Guiyang, China; ^5^Department of Phamaceutical Sciences, School of Pharmacy at Fukuoka, International University of Health and Welfare, Fukuoka, Japan; ^6^Mount Sinai Health System New York, New York, NY, United States

**Keywords:** intracerebral hemorrhage, minimally invasive treatment, hematoma removal, treatment, precision

Intracerebral hemorrhage (ICH) is a life-threatening disease and has the clinical characteristics of high incidence, long treatment cycle, and bleak prognosis, which cause a global health burden. Following the initial irreversible tissue injury, a secondary brain injury induced by hematoma is closely associated with severe neurological deficit. Therefore, early evacuation of the hematoma plays a critical role in alleviating the severity of neurological impairment following ICH. Craniotomy, the traditional surgical option, is traumatic and tends to result in inevitable damage to normal brain tissue. Furthermore, it has neither improved functional prognosis nor lowered the mortality rate when compared with conservative treatment (1, 2). Meanwhile, minimally invasive surgery (MIS), a new operative procedure for ICH, has developed rapidly. MIS is expected to reduce mortality and improve the prognosis of patients with ICH. As professional scholars in the field of ICH research, Prof. Zhouping Tang, John Zhang, Qiang Dong, Guofeng Wu, Yu Hasegawa, and Christopher Paul Kellner were invited to curate the Research Topic “*Precision of Minimally Invasive Surgery for Intracerebral Hemorrhage Treatment*” for *Frontiers in Neurology*.

This Research Topic focuses on recent advances in the precision of MIS for ICH treatment. Twenty-seven manuscripts were submitted and twelve were accepted. A brief description of these research findings follows.

Jiang et al. established an *in vitro* blood clot model to examine the dissolution effect, focusing on the optimal concentration and action time of alteplase (rt-PA) in arterial blood clots derived from patients with ICH. They suggest that a low concentration of rt-PA produces a better dissolution effect and that it functions in a time-dependent manner, reaching a peak at 90 min. This provides new insights into the therapeutic optimization of patients with ICH undergoing MIS.

Hou et al. through meta-analysis, show that the MIS technique could independently reduce the death rate in both a short- and long-term follow-up. In addition, further analysis found that the MIS technique appears to be beneficial when compared to conservative medication. However, the authors did not conduct any subgroup analyses based on different hemorrhage locations. Two in three spontaneous ICH are deep ICH and the remaining third are lobar ICH. Evidence-based medical studies by Akram et al. reveal that no statistical difference was observed between surgery and medical treatment for lobar ICH. Therefore, future studies with larger sample sizes are needed to determine the actual effect of MIS in lobar ICH.

There are two main approaches to MIS: stereotactic hematoma puncture and drainage, and endoscopic hematoma removal. No widely accepted standard is currently available, although various methods of stereotactic aspirations have been established. Wang H. et al. present their single-center experience in the application of an easy, fast, and effective procedure for hematoma evacuation in a total of 45 patients with spontaneous ICH. With the assistance of a location sticker and a brain surgery head frame, the operator could design personalized puncture routes based on the different hematoma locations and volumes. However, because of the limited number of enrolled patients and the lack of a control group, a prospective randomized controlled study was needed to further validate the safety and effectiveness of this approach. Regarding endoscopic evacuation, Hsu et al. share over a decade of experience and outline the progress of MIS for ICH treatment. This is expected to promote the specialization of ICH surgery in the field of MIS. They suggest that MIS, under the guidance of an endoscope for ICH evacuation, is safe and effective. The rehemorrhage ratio and mortality rates are shown to be lower than those of craniotomy. Furthermore, through a retrospective review of 53 patients with ICH undergoing neuroendoscopic surgery, Wu et al. suggest that robot-assisted neuroendoscopic hematoma evacuation, when combined with intracranial pressure monitoring, could improve the curative effect of patients with ICH. This is superior to single neuroendoscopic hematoma evacuation in terms of safety and effectiveness. MIS, when combined with the use of medications, might lessen secondary brain injury following ICH. Ren et al. find that a combination treatment of MIS and deferoxamine could increase perihematomal Claudin-5 and ZO-1 expression levels, reduce blood-brain barrier permeability in the ICH rat model, and improve neurological function. These outcomes may thus prevent secondary brain damage after ICH.

Intraventricular hemorrhage (IVH) adds to the morbidity and mortality of ICH and is an independent predictor of worse outcomes. Zheng et al. in their extensive review, introduce pharmacological catheter- and mechanical-based minimally invasive approaches for ICH and IVH. They firmly believe more clear evidence is expected in the near future to support the use of MIS for ICH treatment. Primary brainstem hemorrhage (PBSH) is the most fatal form of ICH and has a consistently worse prognosis. The management of PBSH is mainly subjected to conservative treatment and surgical procedures are generally not recommended based on current clinical evidence. Chen D. et al. summarize the common prognostic factors and four main surgical options of PBSH. Inconsistent with existing viewpoints, the authors argue that PBSH patients with a score of 2–3 points by a novel grading scale, entitled “the new primary pontine hemorrhage (PPH) score,” seemed to benefit from surgery, although this claim has yet to be demonstrated.

Shen et al. in their original paper, discover that higher levels of fasting blood glucose, neutrophil to lymphocyte ratio, thrombin time at admission, and NIHSS score after surgery were related to symptomatic ICH after endovascular treatment of large vessel occlusion stroke. Moreover, hematoma expansion (HE) indicates a poor prognosis after spontaneous ICH. Hematoma shape-related signs, including the blend sign, spot sign, and island sign, are identified as imaging markers for determining potential individuals who have a high risk of HE among ICH patients (3). In the original paper contributed by Chen Y. et al., the authors assert that the attenuation value of the non-hypodense region of hematoma could also predict HE and the attenuation value of <64 HU was proved to be an appropriate cut-off value of early HE. Post-operative rehemorrhage is one of the most serious complications in patients with ICH undergoing MIS and predicts a more pessimistic prognosis. Postoperative rehemorrhage is therefore seen as an obvious target for medical intervention. Irregular-shaped hematoma (ISH) is a dependable predictor of HE (4). However, few studies have assessed the correlation of ISH with postoperative rehemorrhage in patients with ICH following MIS. To demonstrate the relationship between them, Wang L. et al. carry out a binary logistic regression analysis, which indicates that ISH served as an independent prognostic factor of rehemorrhage after MIS.

Collectively, the current Research Topic provides valuable information on, and insights into, the precision of MIS for ICH treatment. More work is needed to deepen our understanding and grasp of this technology. The use of MIS for ICH treatment should be guided by “stratified decrease of intracranial pressure, liquefaction and drainage of hematoma, and transformation of large hematoma into small one” (5), and eventually lead to the precise treatment of patients with ICH. It is critical to continue to focus on the next steps in promoting the standardization, quantification, precision, and personalization of MIS to facilitate the application of this technology in clinical practice.

## Author contributions

All authors equally contributed to the drafting and writing of the editorial. All authors contributed to the article and approved the submitted version.

## Funding

This work was supported by grants from the National Natural Science Foundation of China (82071330 and 92148206); the Hubei Provincial Department of Science & Technology (2021BCA109).

## Conflict of interest

The authors declare that the research was conducted in the absence of any commercial or financial relationships that could be construed as a potential conflict of interest.

## Publisher's note

All claims expressed in this article are solely those of the authors and do not necessarily represent those of their affiliated organizations, or those of the publisher, the editors and the reviewers. Any product that may be evaluated in this article, or claim that may be made by its manufacturer, is not guaranteed or endorsed by the publisher.
